# A gene expression biomarker for predictive toxicology to identify chemical modulators of NF-κB

**DOI:** 10.1371/journal.pone.0261854

**Published:** 2022-02-02

**Authors:** Katharine L. Korunes, Jie Liu, Ruili Huang, Menghang Xia, Keith A. Houck, J. Christopher Corton

**Affiliations:** 1 Center for Computational Toxicology and Exposure, US Environmental Protection Agency, Research Triangle Park, North Carolina, United States of America; 2 Biology Department, Duke University, Durham, North Carolina, United States of America; 3 National Center for Advancing Translational Sciences, National Institutes of Health, Bethesda, Maryland, United States of America; University of Southern California, UNITED STATES

## Abstract

The nuclear factor-kappa B (NF-κB) is a transcription factor with important roles in inflammation, immune response, and oncogenesis. Dysregulation of NF-κB signaling is associated with inflammation and certain cancers. We developed a gene expression biomarker predictive of NF-κB modulation and used the biomarker to screen a large compendia of gene expression data. The biomarker consists of 108 genes responsive to tumor necrosis factor α in the absence but not the presence of IκB, an inhibitor of NF-κB. Using a set of 450 profiles from cells treated with immunomodulatory factors with known NF-κB activity, the balanced accuracy for prediction of NF-κB activation was > 90%. The biomarker was used to screen a microarray compendium consisting of 12,061 microarray comparisons from human cells exposed to 2,672 individual chemicals to identify chemicals that could cause toxic effects through NF-κB. There were 215 and 49 chemicals that were identified as putative or known NF-κB activators or suppressors, respectively. NF-κB activators were also identified using two high-throughput screening assays; 165 out of the ~3,800 chemicals (ToxCast assay) and 55 out of ~7,500 unique compounds (Tox21 assay) were identified as potential activators. A set of 32 chemicals not previously associated with NF-κB activation and which partially overlapped between the different screens were selected for validation in wild-type and *NFKB1*-null HeLa cells. Using RT-qPCR and targeted RNA-Seq, 31 of the 32 chemicals were confirmed to be NF-κB activators. These results comprehensively identify a set of chemicals that could cause toxic effects through NF-κB.

## Introduction

The transcription factor NF-κB controls the expression of a battery of genes involved in diverse processes including inflammation, immunity, development, and apoptosis [1–5]. NF-κB-mediated pathogenesis has led to interest in understanding the pathogens, stressors, cytokines, and environmental chemicals that affect the NF-κB pathway [6–9]. Because of the relevance of NF-κB dysregulation in human disease, several high-throughput screens (HTSs) have been carried out to identify NF-κB modulators that could be linked to effects on immunomodulation and cancer [10, 11]. The biological significance of these findings has yet to be fully understood for their potential impacts on human health.

There are five known NF-κB family members: NF-κB1 (p50/p105), NF-κB2 (p52/p100, RelA (p65), RelB and c-Rel [12]. NF-κB is present in the cytoplasm of cells in an inactive state as either a homodimer or heterodimer complexed with an inhibitory molecule of the κB family, most commonly IκBα [5]. NF-κB activation through inactivation of IκB can occur through canonical or noncanonical pathways [12]. In the canonical pathway, stimuli activate IκB kinase (IKK) complexes to phosphorylate IκB family members triggering their ubiquitination and subsequent degradation. The degradation of IκB then allows the release and nuclear translocation of active NF-κB leading to alteration of gene expression. Inducers of the canonical pathway include inflammatory cytokines, such as tumor necrosis factor α (TNFα), interleukins, and bacterial products. TNFα binding to the TNF receptor (TNFR) represents one of the most well-studied IKK induction pathways. Toll-like receptor (TLR) agonists, such as lipopolysaccharide (LPS), can also lead to the activation of NF-κB. LPS acts through TLR4 which in turn acts through both adaptor MyD88-dependent and -independent pathways to activate NF-κB. In the MyD88-dependent pathway, interleukin-1 receptor-associated kinase 1 (IRAK1) and IRAK4 phosphorylate TNF receptor-associated factor 6 (TRAF6), which activates IKK. For the MyD88-independent pathway, IKK activation downstream of RIPs and TRAFs is largely mediated by transforming growth beta-activated kinase 1 (TAK1) [13, 14]. In the non-canonical NF-κB pathway, there is selective activation of p100-sequestered NF-κB members, predominantly NF-κB2 p52 and RELB (also referred to as non-canonical NF-κB family members) [4]. In the present study, we focus on identification of chemicals that modulate the canonical NF-κB pathway mediated by NF-κB1, RelA, and c-Rel.

Dysfunction of NF-κB plays a role in oncogenesis by inhibiting apoptosis, stimulating cell proliferation, and affecting inflammation and immunity in ways that create favorable environments for cancer [1]. Misregulation of the NF-κB pathway is also linked to inflammatory diseases such as rheumatoid arthritis and asthma [2–4]. Known environmental factors that are linked to NF-κB activation include cigarette smoke, nanoparticles, asbestos, and lead [6–9]. A high-throughput screen for NF-κB chemical activators was carried out as part of the Environmental Protection Agency (EPA) ToxCast screening program (https://www.epa.gov/chemical-research/toxicity-forecasting) which encompasses ~700 HTS Tier 1 assays representing ~350 molecular targets that have been used to screen more than 3800 chemicals [10, 15]. Another HTS for NF-κB activators was carried out by the National Center for Advancing Translational Sciences (NCATS) as part of the Tox21 screening program (https://tripod.nih.gov/tox21/assays/), an ongoing effort to test the effects of over 10,000 compounds on nuclear receptors, stress response pathways, developmental pathways, and other cellular processes [16]. These high-throughput screens pave the way for understanding the effects of environmentally relevant chemicals on NF-κB. In addition, a screen for NF-κB inhibitors was carried out on ~2800 clinically approved drugs and bioactive compounds from the NIH Chemical Genomics Center Pharmaceutical Collection (NPC) [17] in a NF-κB mediated beta-lactamase reporter gene assay [11] and demonstrated that many currently approved pharmaceuticals have previously unappreciated NF-κB signaling suppression activity.

High-throughput transcriptomic (HTTr) technologies are being increasingly used to screen chemicals in human cell lines. In the EPA ToxCast screening program, HTTr strategies are now being used to replace the battery of individual ToxCast screening assays with targeted sequencing techniques [18] such as TempO-Seq [19]. Compared to individual assays, HTTr technologies have the advantage of examining the effects of environmental chemicals on essentially all pathways operating in cell models, many of which are not examined by the current battery of ToxCast assays. A major challenge is how to interpret the gene expression profiles to identify the molecular targets of chemicals. A number of approaches have been used to interpret the HTTr profiles and these include pathway analysis and comparison to archived profiles of reference chemicals with known bioactivity [20]. Gene expression biomarkers have emerged as an alternative approach to accurately predict specific targets of chemicals. Biomarkers consist of sets of genes known or predicted to be regulated by a particular factor [21]. The biomarker gene expression pattern is compared to gene expression profiles derived from human cells exposed to chemicals using a number of computational techniques that include correlation analysis [22].

Gene expression biomarkers have recently been developed to predict the modulation of a number of transcription factors in human cell lines. Biomarkers for estrogen receptor [1, 23] (Ryan, Chorley et al. 2016) [1] (Ryan, Chorley et al. 2016), androgen receptor [24], metal-induced transcription factor 1 [25], and the oxidant-induced transcription factor Nrf2 [26] have been described. In addition, a biomarker that identifies chemical exposure conditions that lead to DNA damage has been extensively characterized [27, 28] and is currently undergoing review by the Food and Drug Administration to be used as a tool to identify potential DNA damaging agents in human cells. These studies indicate that a methodical analysis of gene expression profiles of reference chemicals and appropriate genetic perturbations will eventually lead to a battery of highly predictive biomarkers that can be used to interpret HTTr data streams [21]. The large quantity of gene expression data that already exists in commercial and public repositories will provide *in silico* high-throughput identification of chemical agents that activate or suppress human molecular targets including NF-κB. Approaches to assess NF-κB modulation using HTTr data have not been previously described.

In the present study, we developed procedures for predicting NF-κB perturbation in HTTr data. We constructed a gene expression biomarker that accurately predicts NF-κB modulation after exposure to immunomodulatory factors and chemicals. We used the biomarker to screen a library of microarray profiles from cells treated with ~2600 organic chemicals to identify modulators of NF-κB. There were 215 and 49 chemicals that were identified as putative or known NF-κB activators or suppressors, respectively using our gene expression biomarker approach. NF-κB activators were also identified using two high-throughput screening assays; 165 out of the ~3,800 chemicals screened in the ToxCast assay and 55 out of ~7,500 unique compounds screened in the Tox21 program were identified as potential activators. We examined a set of 32 putative activating chemicals not previously recognized as NF-κB activators by comparing expression patterns of NF-κB-regulated genes in wild-type and *NFKB1*-null cells. We validated 31 out of the 32 chemicals as NF-κB activators, providing a set of chemicals that could potentially activate NF-κB-associated effects in animals and humans. Overall, our approach greatly expands the number of environmentally relevant chemicals that putatively activate NF-κB.

## Results

### Identification of NF-κB biomarker genes

**Fig 1:**
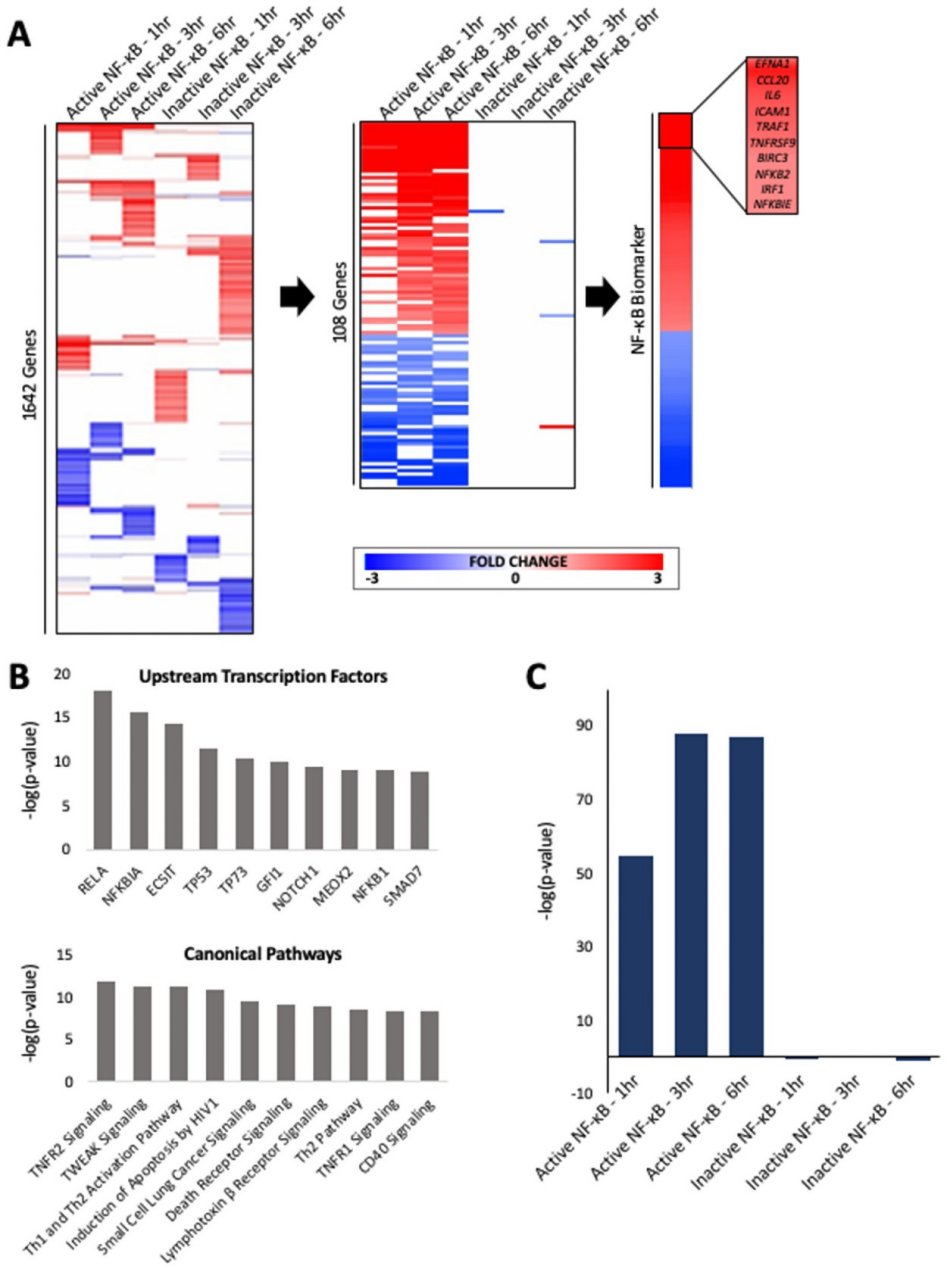
Building and characterizing the NF-κB biomarker. (A) Gene expression profiles of the six biosets used to construct the NF-κB biomarker. (Left) The fold-changes of the statistically significant genes from the Tian *et al*. (2005) study are shown after one-dimensional clustering of genes. (Center) Expression of the genes that make up the biomarker after TNFα treatment in wild-type and IκB-expressing cell lines are shown. (Right) The fold-change values averaged across treatments in wild-type cells yielded the 108 gene NF-κB biomarker, with the names of the top 10 genes shown. (B) Ingenuity Pathway Analysis of biomarker genes. (Top) Transcription factors predicted to regulate the biomarker genes using the upstream regulator analysis. (Bottom) Canonical pathways that significantly overlap with the biomarker genes. Biomarker genes were compared to the genes in the canonical pathway lists from IPA. (C) Comparison of the biomarker to the biosets used to construct the biomarker. The -log(p-value)s of the pairwise correlations are in the same order as those in A (middle).

A set of biomarker genes was identified that could be potentially used to predict modulation of NF-κB activity in transcript profiles. We utilized a previously published study in which wild-type HeLa cells and HeLa cells which overexpressed the NF-κB inhibitor IκB were treated with TNFα for 1h, 3h, and 6h [29]. The expression of statistically filtered and clustered genes in each treatment group is shown in Fig 1A **(left)**. A straightforward weight of evidence approach was used to identify genes which exhibited consistent expression changes after TNFα exposure at the different time points in the wild-type but not the IκB-expressing cells (specific criteria described in Methods). The behavior of the resulting 108 genes across the 6 treatment groups is shown in Fig 1A **(middle)**. The resulting biomarker consisted of 63 upregulated genes and 45 downregulated genes with fold-change values derived from the average fold-change across the three time points in wild-type cells (Fig 1A **(right))**. The full list of biomarker genes is found in **S1 Table in** S1 File. Notably, many of the highest ranking genes in the biomarker are known direct targets of NF-κB including *IL6* [30], *ICAM1* [31], and *IRF1* [32] and have well-characterized functions within the NF-κB regulatory network.

### Analysis of pathways overlapping with biomarker genes

We identified pathways enriched in biomarker genes using the canonical pathway and upstream analysis functions in Ingenuity Pathway Analysis (IPA) (Fig 1B). Several of the top upstream regulators predicted by the analysis were transcription factors and included RELA, NFKB1A, and NFKB1. The analysis identified the TNFR2 and TWEAK signaling pathways as the top canonical pathways, with 26.7% (p = 1.36 x 10^−12^) and 22.9% (p = 5.37 x 10^−12^) of the genes overlapping, respectively. The complete set of IPA results are found in **S2** and **S3 Tables in** S1 File.

### Comparison of the biomarker to individual biosets used to create the biomarker

The rank-based Running Fisher algorithm was used to determine the pair-wise correlations between the biomarker and the biosets used to construct the biomarker. Statistically significant correlations were defined as those with a |-log(*p*-value)| ≥ 4 [25, 26] where positive values indicated a condition that led to activation of NF-κB, while negatively correlated biosets were predicted to be conditions that led to inhibition of NF-κB. The three biosets from TNFα-treated wild-type cells were positively correlated with the biomarker with very high -Log(p-value)s, as expected (Fig 1C). In contrast, the biosets from the TNFα-treated IκB-expressing cells did not exhibit significant correlation with the biomarker due to the fact that only 0–3 genes overlapped between the biosets and the biomarker. This observation was preliminary evidence that the biomarker correlations with biosets are driven by NF-κB-dependent activity, as discussed in detail below.

### Predictive accuracy of the biomarker

**Fig 2:**
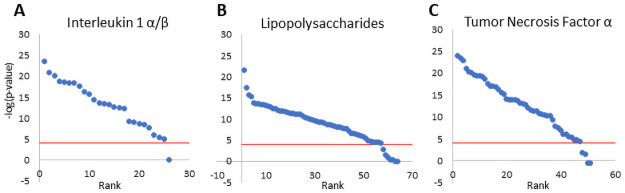
Assessment of the accuracy of the biomarker in predicting NF-κB activation. The sensitivity of the biomarker was determined using studies from cells expected to exhibit NF-κB activation after exposure to (A) interleukin 1α/β, (B) lipopolysaccharides, and (C) tumor necrosis factor α. For each factor, the -log(p-values) for the studies were rank-ordered. The red line shows the cutoff for statistical significance (-log(p-value) = 4).

In the human gene expression compendium, there were 46, 107, and 88 biosets in which interleukin 1α/β (IL1α/β), lipopolysaccharide (LPS), or TNFα, respectively were used to treat various human cell lines under conditions expected to activate NF-κB. We tested the sensitivity of predicting NF-κB activation by determining how many of the biosets exhibited a -log(p-value) ≥ 4. The sensitivities for IL1α/β, LPS, and TNFα were 93.5%, 85%, and 89.8%, respectively indicating the biomarker is very predictive in identifying treatments that lead to NF-κB activation. This consistency was despite the fact that the biosets were derived from a heterogeneous set of experiments generated in different labs on different microarray platforms. There was no consistent basis for why some of the treatments lacked significant correlation. However, we did note that for some of the studies evaluated, NF-κB activation was observed only during a narrow window of exposure times which varied between studies. With this in mind, we reevaluated the sensitivity predictions for each study by grouping the biosets within that study. If any bioset from that study examining the same factor was positive for NF-κB activation, then the study was counted as a positive. Performing the analysis in this manner slightly improved the sensitivities to 91.5% in 26 studies using IL1α/β, 64 studies using LPS, and 51 studies using TNFα (Fig 2). Overall, our methods were very accurate at identifying true positives from a range of treatments from heterogeneous studies that led to activation of NF-κB.

The specificity of the biomarker was also determined using the biosets derived from cells treated with factors not expected to activate NF-κB. These included IL2, IL3, IL4, IL6, IL12, interferon (IFN)α, and IFNβ. There were 208 biosets evaluated. Exposure to cytokines IL2, IL3, IL4, IL6, and IL12 did not lead to consistent activation or inhibition of NF-κB; only 12 of the 208 biosets were positive for NF-κB activation (**S1A Fig in** S2 File). Likewise, treatment with IFNα or IFNβ did not lead to consistent effects on NF-κB (**S1B Fig in** S2 File). Four of the six IFN positives from cells treated with IFNβ came from the same study using hepatoma HuH-7 cells (GSE48400). There are reports that IFNγ treatment activates NF-κB through both the canonical and noncanonical pathways that may be dependent on cellular context [33, 34]. Eighteen of the 47 biosets from cells treated with IFNγ led to activation. (**S1C Fig in** S2 File). Using the data from all cytokines except IFNγ, our approach resulted in a specificity of 94.2%. Performing the analysis on a study basis similar to that described above gave a specificity of 89.1%. Combining the predictions from the sensitivity and specificity calculations gave a balanced accuracy of 91.3% for individual comparisons and 90.3% on a study level. Thus, the biomarker is a reliable predictor of activation through the canonical pathway but not necessarily through other immunomodulatory pathways. The biosets used in the analysis are found in **S4 Table in** S1 File.

### The biomarker identifies diverse conditions that activate NF-κB

**Fig 3:**
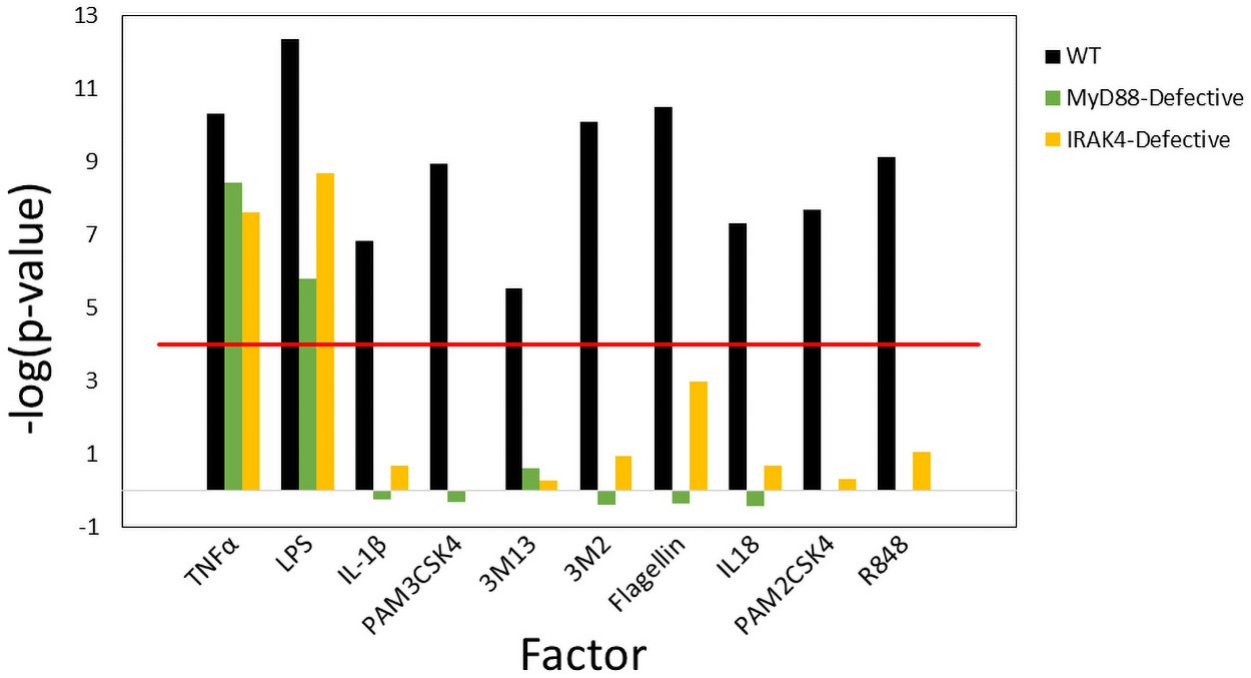
The biomarker predicts activation by Toll-like receptor and interleukin receptor agonists. Significance of the pairwise comparisons between microarray profiles and the biomarker are shown for experiments in which whole blood from normal, *MyD88*-defective, or *IRAK4*-defective patients was stimulated for 2 hours with the indicated agonists (data from GSE25742 [35].

We determined if the biomarker would respond in a predictable manner to a diverse array of Toll-like and Interleukin 1 receptor agonists. Whole blood from normal, *MyD88*-defective, or *IRAK4*-defective patients was stimulated for 2 hrs in vitro with agonists encompassing most TLRs (PAM3 (TLR1/2), PAM2 (TLR2/6), LPS (TLR4), Flagellin (TLR5), 3M2 (TLR7), 3M13 (TLR8), R848 (TLR7/8), and IL-1 receptors (IL-1β, IL-18) along with the positive control, TNFα (data from [35]; GSE25742). Comparison of the microarray profiles from each of the 33 comparisons to the biomarker is shown in Fig 3 (biosets are listed in **S5 Table in** S1 File). TNFα and LPS showed significant activation (-log(p-value) ≥ 4) in wild-type cells and both appeared to be partially dependent on *MyD88* and *IRAK4* evidence by the decrease in the significance in the correlation. LPS activates NF-κB through both *MyD88*/*IRAK4* and TRAF6, another component of the *IRAK4* complex [36]. It is thought that TNFα can activate NF-κB through *MyD88*- and *IRAK4*-independent pathways [37]. Activation of NF-κB by IL18, IL1β, PAM3, PAM2, flagellin, 3M2, R848, and 3M13 was dependent on both *MyD88* and *IRAK4* consistent with previously characterized signaling networks. Thus, our biomarker approach identified treatments that are known to activate NF-κB in a manner that reflects well-characterized dependencies on *MyD88* and *IRAK4*.

We examined the effects of other factors known to affect NF-κB signaling. The cytokine tumor necrosis factor-like weak inducer of apoptosis (TWEAK) which binds the receptor FN14 leads to activation of NF-κB through both canonical and noncanonical pathways [38]. All 11 of the biosets in which three cell types were exposed to TWEAK exhibited NF-κB activation (**S1D Fig in** S2 File). Treatment with antibodies against CD3 and CD28 which activate resting T-cells activate NF-κB through a noncanonical pathway [39]. More than half of the 33 biosets derived from cells treated with anti-CD3/anti-CD28 exhibited NF-κB activation (**S1E Fig in** S2 File).

### Screen for chemical modulators of NF-κB

**Fig 4:**
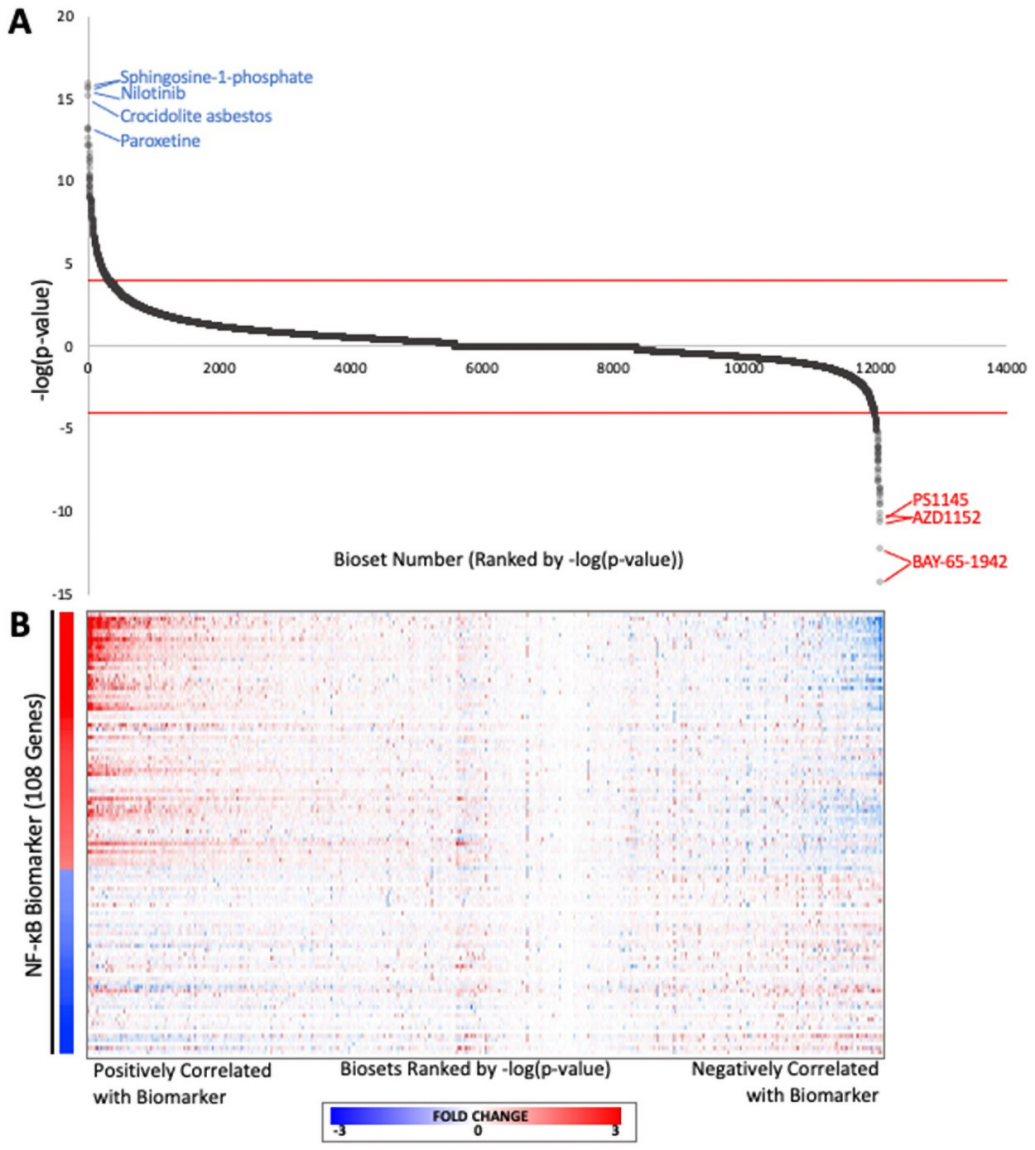
Screening a human microarray compendium for NF-κB chemical modulators. (A) The -log(*p*-values) for correlations between the NF-κB biomarker and each of the biosets representing cells exposed to individual chemicals. Biosets were rank ordered by -log(*p*-value) of the correlation between the biomarker and the bioset. The cutoffs for statistical significance are shown with dashed lines. The biosets with -log(*p*-value) ≥ 4 were considered positively correlated while the biosets with -log(*p*-value) ≤ -4 were considered negatively correlated with the biomarker. The top five biosets predicted to be activators or inhibitors are shown with the chemical names. (B) Gene expression changes for NF-κB biomarker genes across the biosets evaluating chemical-induced changes in gene expression. The NF-κB biomarker fold-change values are shown on the left.

We performed an *in silico* screen to identify novel organic chemicals that modulate NF-κB. We compared the biomarker against a human expression compendium [22] consisting of 12,061 biosets representing human cell lines exposed to 2,672 chemicals. The ranked -log(*p*-value)s for individual biosets are shown in Fig 4A. The statistically filtered fold-change values of the biomarker genes for the corresponding biosets are shown in Fig 4B. For those biosets that were positively correlated with the biomarker, the expression of the biomarker genes was remarkably similar to the biomarker itself in terms of direction of change as well as relative gene rankings especially for those genes that were positively regulated. For those biosets that were negatively correlated, the expression of the biomarker genes generally exhibited opposite expression compared to the biomarker genes. The analysis identified 351 biosets representing 215 chemicals that were positively correlated and 83 biosets representing 49 chemicals that were negatively correlated with the NF-κB biomarker (**S6 Table in** S1 File). The remaining 11,627 biosets were not significantly correlated with the biomarker in either direction.

### Characterization of chemicals predicted to activate NF-κB

**Fig 5:**
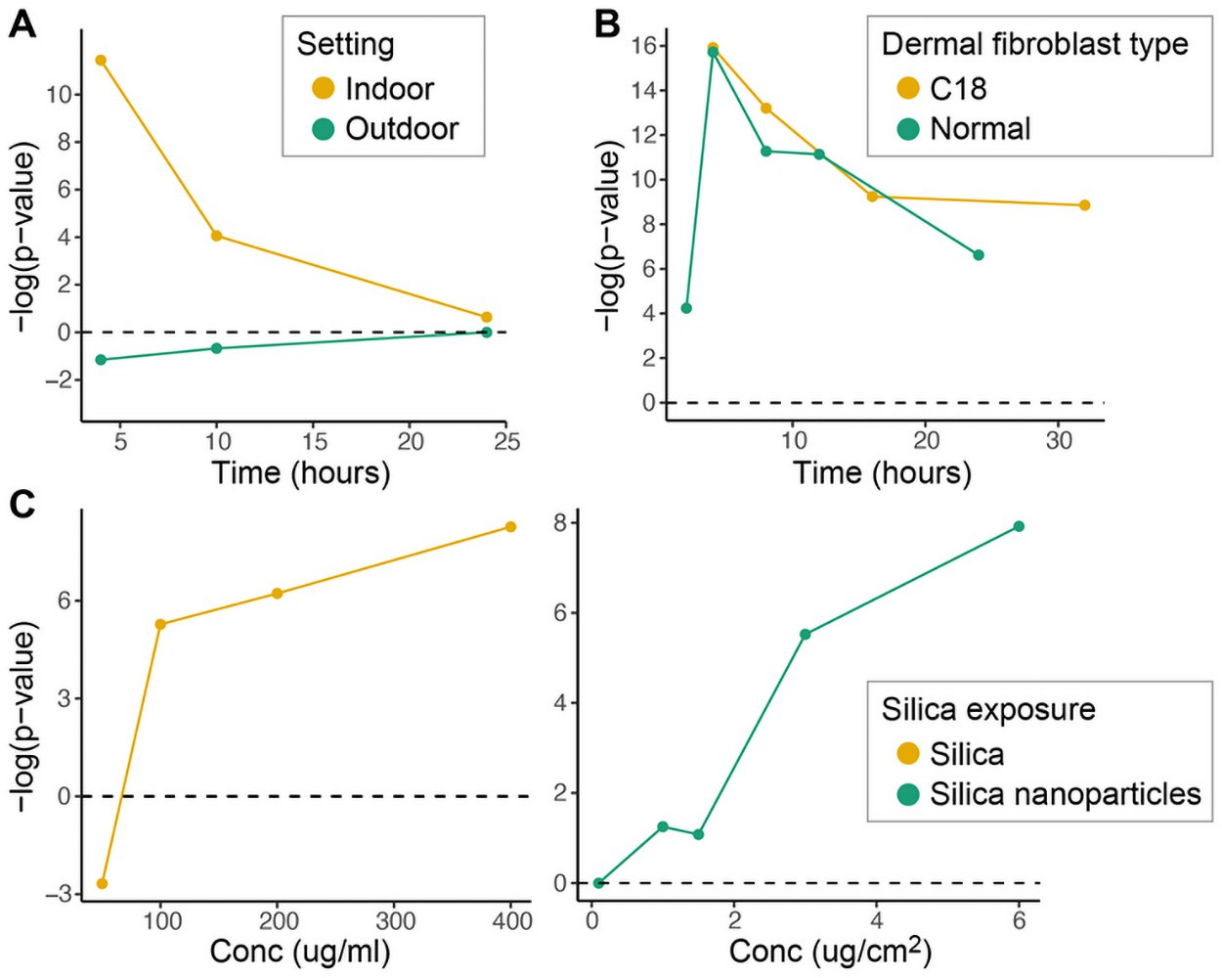
Characterization of NF-κB activators. (A) Time-dependent changes in NF-κB activation after exposure to particulate matter (PM)10 isolated from either indoor air from classroom settings (indoor) or outdoor air in bronchial epithelial BEAS-2B cells (from GSE34607). (B) Time-dependent changes in activation of NF-κB by 3uM sphingosine-1-phosphate in dermal fibroblasts from primary normal human dermal fibroblasts (normal) or C18 dermal fibroblasts (C18) (data from GSE56308). (C) Concentration-dependent increase in NF-κB activity after exposure to silica (left; from GSE30213 and GSE30200) and silica nanoparticles (right; from GSE63806) in A549 cells.

The top 20 ranking biosets that resulted in NF-κB activation are shown in Table 1. The biosets included five from one study examining the effects of sphingosine-1-phosphate (S1P) on dermal fibroblasts. NF-κB is known to be activated by extracellular S1P via S1P2 receptors and G_i_ protein signaling [2, 40] (Blom, Bergelin et al. 2010) [2]. There is evidence that NF-κB is activated by chemicals in the top 20 biosets including crocidolite asbestos [3, 41] (Janssen, Driscoll et al. 1997) [3] (Janssen, Driscoll et al. 1997), nickel chloride [42], and curdlan [4, 43] (Rand, Robbins et al. 2013) [4]. Silicon dioxide and particulate matter are discussed below. R848 is a TLR7 agonist that activates NF-κB [44]. A number of datasets were examined in detail to determine if the biomarker detects time- and concentration-dependent NF-κB activation. Fig 5A shows the time-dependent changes in NF-κB activation after exposure to particulate matter with diameters of 10um or smaller (PM10) isolated from either indoor air from classroom settings (indoor) or outdoor air examined in bronchial epithelial BEAS-2B cells (from GSE34607). The authors noted that indoor PM10, compared to outdoor PM10, induced more inflammatory and allergenic reactions, and accelerated blood coagulation. Outdoor PM10 induced a different pattern of gene expression that included detoxifying enzymes [45]. Fig 5B shows the time-dependent changes in activation of NF-κB by 3uM S1P in primary normal human dermal fibroblasts (normal) or patient C18 dermal fibroblasts (C18) (data from GSE56308). The authors of the study noted a strong correlation between S1P exposure and a subset of genes involved in inflammation indicating a role for S1P in immune activation in systemic sclerosis, a progressive fibrotic disease of unknown etiology [46]. Fig 5C shows the concentration-dependent increase in NF-κB activity after exposure to silica from two companion studies (left; from GSE30213 (50 ug/mL) and GSE30200 (100, 200, 400 ug/mL)) and silica nanoparticles (right; from GSE63806) in lung epithelial A549 cells. While the authors noted that silica exposure leads to inflammation, they did not characterize NF-κB activation [47, 48]. The full list of biosets describing chemicals with significant hits from the biomarker screen is found in **S6 Table in** S1 File.

**Table 1:** The 20 biosets with the highest correlation to the NF-κB biomarker.

Bioset Name	Chemical	Cell type	Public ID	Time of exposure (hrs)	Conc (in uM unless otherwise indicated)	NF-κB biomarker (-Log(p-value))
Dermal fibroblasts (C18)—3uM sphingosine-1-phosphate treated 4hr _vs_ untreated_GPL6480	Sphingosine 1-phosphate	Dermal fibroblasts	GSE56308	4	3	15.9
Dermal fibroblasts (NHDF)—3uM sphingosine-1-phosphate treated 4hr _vs_ untreated_GPL6480	Sphingosine 1-phosphate	Dermal fibroblasts	GSE56308	4	3	15.7
Lung cancer A549 cell line + Nilotinib 30000nM for 4hr _vs_ control	Nilotinib	A549	E-TABM-585	4	30	15.6
Primary mesothelial HPM3 cells, pleural + 5ug/cm2 crocidolite asbestos for 8hr _vs_ unexposed	Asbestos, crocidolite	HPM3	GSE63966	8	5ug/cm2	15.2
Paroxetine_20uM_24hr_Chen	Paroxetine	MCF7	PMID: 24496634	24	20	13.3
Macrophages monocyte-derived 10uM nutlin-3 treated 2hr _vs_ DMSO	Nutlin 3	Monocyte-derived macrophages	GSE43596	2	10	13.2
Dermal fibroblasts (C18)—3uM sphingosine-1-phosphate treated 8hr _vs_ untreated_GPL6480	Sphingosine 1-phosphate	Dermal fibroblasts	GSE56308	8	3	13.2
HUVEC cells + 1.5 mM nickel chloride for 5hr _vs_ control	Nickel chloride	HUVEC	GSE4852	5	1500	12.6
Bronchial epithelial BEAS-2B cells treated 4hr with 10ug/ml PM10—indoor _vs_ outdoor	Particulate matter	BEAS-2B	GSE34607	4	10ug/ml	12.2
Huh7 hepatocarcinoma cells 10uM GENK treated for 4hr _vs_ DMSO control	Genkwanin	HuH-7	GSE39002	4	10	12.2
A549 lung carcinoma cells grown in presence of 10nM geldanamycin (IC20) 48hr _vs_ vehicle	Geldanamycin	A549	GSE26525	48	0.01	12.2
Monocyte derived dendritic cells + TLR4 agonist R848 2hr _vs_ untreated	R848	Monocyte-derived dendridic cells	GSE2706	2	2.5 μg/ml	11.8
HEK293 cells overexpression p65-S547A mutant—etoposide treated 8hr _vs_ untreated	Etoposide	HEK293	GSE33990	8	Not reported	11.5
Bronchial epithelial BEAS-2B cells—4hr 10ug/ml indoor PM10 _vs_ 4hr untreated	Particulate matter	BEAS-2B	GSE34607	4	10ug/ml	11.4
Dermal fibroblasts (NHDF)—3uM sphingosine-1-phosphate treated 8hr _vs_ untreated_GPL6480	Sphingosine 1-phosphate	Dermal fibroblasts	GSE56308	8	3	11.3
Dermal fibroblasts (NHDF)—3uM sphingosine-1-phosphate treated 12hr _vs_ untreated_GPL6480	Sphingosine 1-phosphate	Dermal fibroblasts	GSE56308	12	3	11.1
Macrophages treated 6hr with 10ug curdlan _vs_ untreated controls	Curdlan	Macrophage	GSE32282	6	10ug/ml	10.8
Hepatocellular carcinoma hepatocyte HepaRG cells + 6hr 100nM vinblastine _vs_ DMSO	Vinblastine	HepaRG	GSE69851	6	100	10.4
Lung epithelium A549 cells 200ug/ml silica treated from 24hr _vs_ untreated	Silicon dioxide	A549	GSE30215	24	0.1	10.4
HL60 cells + amphotericin B, 4.4uM _vs_ DMSO vehicle	Amphotericin B	HL-60	GSE5258	6	4.4	10.3

### Characterization of chemicals predicted to suppress NF-κB

**Fig 6:**
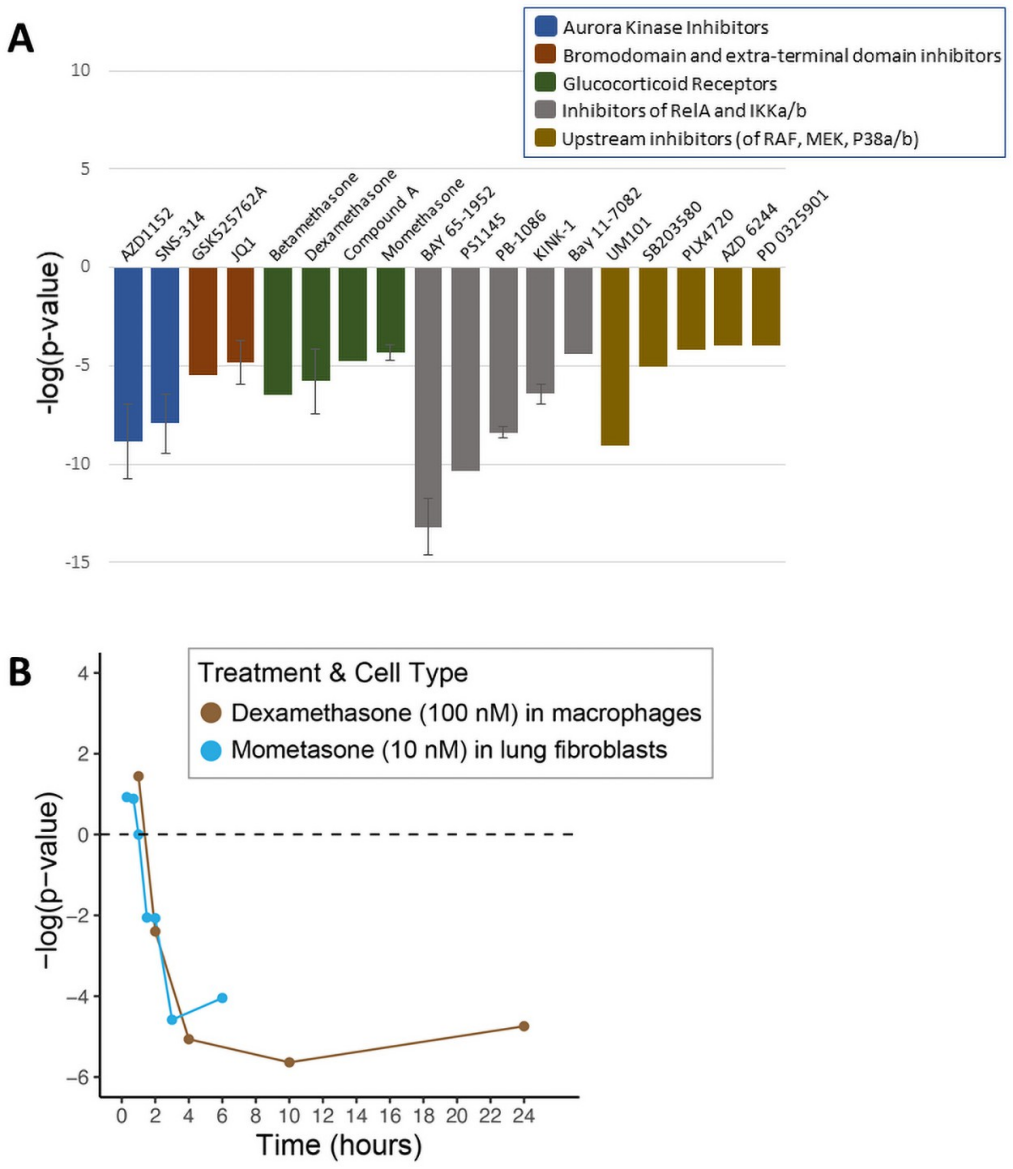
Characterization of NF-κB inhibitors. (A) The -log(p-values) representing the correlation between the NF-κB biomarker and each of the 19 putative NF-κB inhibitors that fell into the five major functional categories of inhibitors (from 49 total biosets). Error bars are shown when multiple biosets assessed a single chemical. (B) Time-dependent NF-κB suppression by 10 nM mometasone in lung fibroblasts (from GSE30242) and 100 nM dexamethasone in macrophages (from GSE61880).

The chemicals predicted to suppress NF-κB fell into a number of groups based on their known molecular target (Table 2). There were six compounds that act as inhibitors of three components of NF-κB signaling. Fig 6A shows that the biomarker predicted NF-κB suppression after exposure to inhibitors of RelA (PB-1086), IKKα (BAY 11–7082), and IKKβ (KINK-1, Bay 65–1942, PBS1145). The second group included two chemicals (AZD1152, SNS-314) in 8 biosets that act as aurora kinase inhibitors. Aurora kinases play a crucial role in cellular division by controlling chromatid segregation [49]. The third group includes four chemicals (AZD 6244, PD0325901, PLX4720, SB203580) that act as inhibitors of overlapping signaling components RAF, MEK, and p38α/β [50, 51]. The fourth group includes two chemicals (JQ1, GSK525762A) that act as bromodomain (BRD) and extra terminal protein (BET) inhibitors. Members of the BET subfamily of proteins play important roles in cell-cycle control and transcription, and recent evidence has established a link between BET proteins and NF-κB-mediated inflammatory response [52]. Lastly, there were 169 biosets which were derived from cells treated with four glucocorticoid receptor agonists (compound A, betamethasone, dexamethasone, mometasone furoate). GR agonists are well known to suppress inflammatory responses through NF-κB [53, 54]. Fig 6B shows two examples of the time-dependent suppression of NF-κB by 10 nM mometasone in lung fibroblasts (from GSE30242) and 100 nM dexamethasone in macrophages (from GSE61880).

**Table 2:** Predicted inhibitors of NF-κB identified using the biomarker screening approach. Forty-nine chemicals predicted to inhibit NF-κB were identified. Shown are experimental conditions that led to significant correlation*.

PREDICTED NF-κB INHIBITOR	CELL LINE(s)	TREATMENT TIME(s)	TREATMENT DOSE(s)	DATA SOURCE(s)
**Inhibitors of RelA, IKKa/b**				
BAY 11–7082	HCMV-infected monocytes			GSE9601
Bay 65–1942	MCF10A cells	4hr		GSE33403
KINK-1	A375 melanoma cells	12hr, 24hr	5uM	GSE8772
PB-1086	Lung cancer 11–18 cells		5uM	GSE65420
PS1145	Lung cancer A549 cells	1.5hr	10uM	GSE34329
**Aurora Kinase Inhibitors**				
AZD1152	Melanoma 1205Lu cells	6hr, 12hr, 18hr, 24hr	0.5uM	GSE38466
SNS-314	Melanoma 1205Lu cells	6hr, 12hr, 18hr, 24hr	0.05uM	GSE38466
**Inhibitors that act upstream of NF-κB on RAF, MEK, and P38a/b**				
AZD 6244	Melanoma Me13 cells	8hr	0.1uM	GSE59882
PD 0325901	Melanoma Me23682 cells	24hr	5nM	GSE34686
PLX4720	Melanoma Me13 cells	8hr	0.5uM	GSE59882
SB203580	Primary cells—hepatocytes		10uM	GSE76098
UM101	Primary cells—lung	1hr	100uM	GSE93330
Doxycycline	Whole Blood—Leukocytes			GSE63085
**Bromodomain and Extra Terminal Domain Inhibitors**				
GSK525762A	Prostate carcinoma PC-3 cells	24hr	10uM	GSE56352
JQ1	Primary Cells—Osteoblasts, Umbilical vein endothelial cells; Prostate cancer PC3 cells; Prostate RWPE cells	2hr, 24hr	250nM, 25ng/mL, 500nM	GSE82289; GSE53999; GSE55063
**Glucocorticoid Receptor Agonists**				
2-(4-acetoxyphenyl)-2-chloro-N-methyl-ethylammonium chloride	Breast adenocarcinom MDA-MB-231 cells	2hr	10uM	GSE56022
Betamethasone	Primary tissue—skin			GSE32473
Dexamethasone	U-2 OS Cell Line; Primary Cells—Leukocytes, Macrophages, Dendritic Cells, Lung Cells; Blood Fraction; MDA-A1 Cell Line	2hr, 4hr, 6hr, 10hr, 8d, 24d	100nM, 1uM	GSE46448; GSE50012; GSE33135; GSE61880; GSE45407; GSE56022; GSE56017; GSE34313
Mometasone	Primary Cells—Fibroblasts	3hr, 6hr		GSE30242
**Miscellaneous or Unknown Mechanisms**				
Tofacitinib	Primary tissue—scalp		5mg	GSE80688
Imatinib	CD34+ hematopoietic stem cells			GSE12211
Simvastatin	Primary cells—hepatocytes	24hr	30uM	TG-GATES
Formoterol	Primary Cells—Fibroblasts	6hr		GSE30242
Givinostat	HDLM-2 Cell Line	24hr	100nM	GSE31060
Etanercept	Primary Cells—Leukocytes			GSE36177
Infliximab	Primary Tissue—RA synovial tissue			E-TABM-104
2-hydroxypropyl-beta-cyclodextrin	Cervical cancer HeLa cells	3hr	2p HBCD	E-TABM-599
2-nitrofluorene	TK6 Cells	7hr		Yauk et al study
Aeb071	Mino Cell Line	3hr	2.5uM	GSE42549
Benzene	B lymphoblast TK6 cells	24hr	0.01mm	GSE87005
Carbon black	Primary cells—epithelium			GSE41178
Carboplatin	Xenograft			GSE55399
Erlotinib	Lung cancer 11–18 cells		100nM	GSE65420
Glucose	HEK293 cells	7d	450 mg/dl	GSE15575
Hydrogen peroxide	Primary Cells—T-lymphocytes	4hr		GSE6607
Mesalamine	Primary cells—Mucus membrane	6hr	50nM	GSE46451
Metformin	THP-1 Cell Line	48hr	2mM	GSE51803
Mln4924	Chronic lymphocytic leukemia B cells	24hr	1uM	GSE44864
Nanotubes, carbon	Primary cells—respiratory epithelium			GSE41178
Nickel-sulfate	Primary tissue—skin	7hr		GSE6281
N-octanoyldopamine	HUVECs	24hr	100uM	GSE34059
Phenethyl isothiocyanate	Primary cells—hepatocytes	48hr	25uM	GSE20479
Pimecrolimus	Primary tissue—skin			GSE32473
Risperidone	SK-N-SH neuroblastoma cells	6hr	10uM	GSE36678
Rituximab	REC-1 Cell Line		10ug/mL	GSE54169
Rosiglitazone	Primary cells—macrophages	72hr		GSE16385
Sulforafan	PC-3 Cell Line	6hr	15uM	GSE48812
Tobacco smoke	Primary cells—alveolar macrophages; Primary tissue—bronchi			GSE13931; GSE37147
Tolvaptan	Primary cells—hepatocytes	72hr	50uM	GSE99878

*Experimental conditions (length of exposure, concentration) are shown here only when this information was clearly presented in the bioset description. Additional details can be found using the indicated accession information.

### Identification of putative NF-kB activators in high-throughput screens

In an effort to comprehensively identify NF-κB modulators, large sets of organic compounds were screened in two NF-κB HTS assays. The first screen conducted by NCATS as part of the Tox21 screening program was carried out using a beta-lactamase reporter gene under control of a NF-κB response element in the human cervical cancer cell line, ME-180. Out of ~7,500 chemicals originally examined, there were 55 unique compounds identified as putative activators, and 26 of these activators were selected for further analyses (**S7 Table in** S1 File). Notably, three compounds represented by four biosets overlapped as hits in both the Tox21 data and in our screen of our microarray compendium. These three compounds (mitoxantrone, thioridazine, vincamine) were examined further, as discussed below.

The second high-throughput screen was carried out as part of the ToxCast screening program by Attagene (under contract to the EPA). In this assay, a reporter gene was under control of a NF-κB response element in the human hepatocyte cell line HepG2. A total of 3,806 samples were screened and 165 were identified as potential activators (**S8 Table in** S1 File).

### Confirmation of the NF-κB Activators

**Fig 7:**
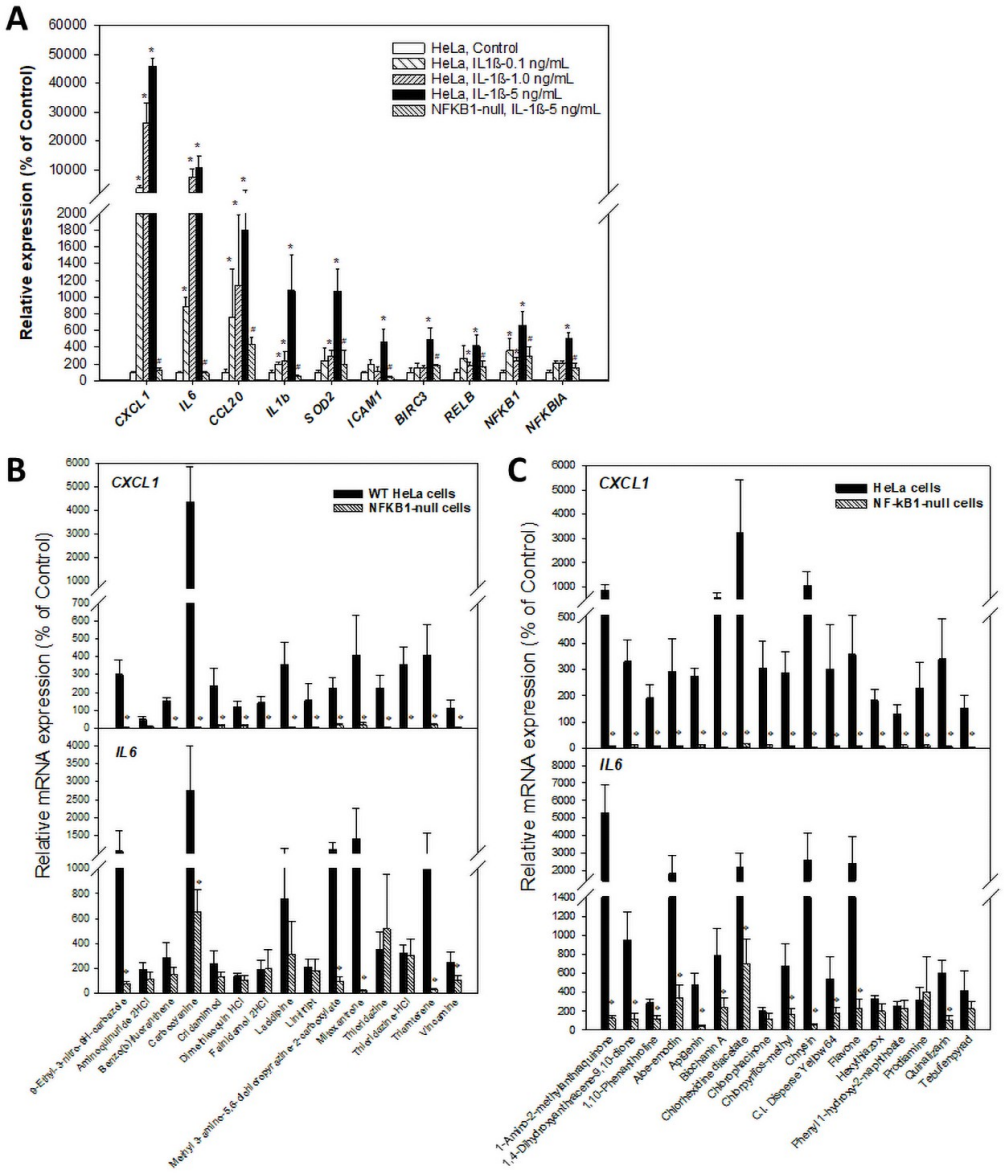
Expression of NF-κB biomarker genes in wild-type and *NFKB1*-1-null cells. Wild-type and *NFKB1*-null HeLa cells were treated with IL1β or the indicated chemicals for 6 hrs and expression of the NF-κB-responsive gene *CXCL1* or several NF-κB biomarker genes were examined by RT-qPCR. (A) Expression changes of NF-κB-responsive genes are diminished or abolished in *NFKB1*-null cells. *Indicates significant difference between treated and control wild-type cells; p-value < 0.05. ^#^ Indicates significant difference between treated wild-type and treated *NFKB1*-null cells; p-value < 0.05. (B) Changes in the expression of *CXCL1* and *IL6* genes after exposure to 15 Tox21 chemicals. (C) Changes in the expression of *CXCL1* and *IL6* genes after exposure to 17 ToxCast chemicals.

We selected a total of 32 chemicals predicted to activate NF-κB from the HTS assays or the biomarker screen (Table 3). The ability of these 32 chemicals to activate NF-κB-dependent genes in wild-type and *NFKB1*-null HeLa cells was tested by RT-qPCR. Expression of NF-κB biomarker genes was first examined in cells after exposure to IL1β for 6 hrs. We first examined a number of genes that were in the biomarker (Fig 7A). In addition, we examined the *CXCL1* gene that was highly induced by TNFα only at one hr in wild-type but not IkB-overexpressing cells [29]. Most genes showed concentration-dependent increases in expression in wild-type cells (Fig 7A). In contrast in the *NFKB1*-null cells, there was little if any increase in expression of the biomarker genes at the highest concentration of IL1β used. Given that HTTr screening efforts at EPA have utilized the MCF7 cell line [5], we confirmed that most of the biomarker genes were responsive to TNFα or IL1β in MCF7 cells (**S2 Fig in** S2 File) indicating that this cell line would be an appropriate HTTr model for identification of chemicals that modulate NF-κB.

**Table 3:** Activators of NF-κB identified by HTS assays examined by RT-qPCR. There were 32 organic chemicals predicted to activate NF-κB selected for further study.

Chemical	CASN	HTS Assay	AC50 (uM)	EFFICACY (fold increase)	Identified using the NF-κB Biomarker	Data Source	Validated as an NF-κB Activator in HeLa Cells by RT-qPCR^1^
1,10-Phenanthroline	66-71-7	ToxCast	77.05706	3.163462	NA	NA	Yes
1,4-Dihydroxyanthracene-9,10-dione	81-64-1	ToxCast	2.125469	1.86884	NA	NA	Yes
1-Amino-2-methylanthraquinone	82-28-0	ToxCast	33.57287	2.952916	NA	NA	Yes
Aloe-emodin	481-72-1	ToxCast	9.564654	2.112953	NA	NA	Yes
Apigenin	520-36-5	ToxCast	24.31648	6.368289	NA	NA	Yes
Biochanin A	491-80-5	ToxCast	128.6124	3.190983	NA	NA	Yes
C.I. Disperse Yellow 64	10319-14-9	ToxCast	26.37337	2.037707	NA	NA	Yes
Chlorhexidine diacetate	56-95-1	ToxCast	30.4311	4.095707	NA	NA	Yes
Chlorophacinone	3691-35-8	ToxCast	31.79655	4.897706	NA	NA	Yes
Chlorpyrifos-methyl	5598-13-0	ToxCast	9.022388	1.603474	NA	NA	Yes
Chrysin	480-40-0	ToxCast	11.41565	2.688455	NA	NA	Yes
Flavone	525-82-6	ToxCast	71.15899	5.433016	NA	NA	Yes
Hexythiazox	78587-05-0	ToxCast	26.59593	2.467522	NA	NA	Yes
Phenyl 1-hydroxy-2-naphthoate	132-54-7	ToxCast	81.67663	1.956113	NA	NA	Yes
Prodiamine	29091-21-2	ToxCast	34.63778	2.925536	NA	NA	Yes
Quinalizarin	81-61-8	ToxCast	27.32025	2.446469	NA	NA	Yes
Tebufenpyrad	119168-77-3	ToxCast	0.804216	2.914699	NA	NA	Yes
9-Ethyl-3-nitro-9H-carbazole	86-20-4	Tox21	61.64481	12.7423	NA	NA	Yes
Aminoquinuride dihydrochloride	5424-37-3	Tox21	22.14763	15.76857	NA	NA	No
Benzo(b)fluoranthene	205-99-2	Tox21	54.941	58.50674	NA	NA	Yes
Carbocyanine	605-91-4	Tox21	19.73909	38.73972	NA	NA	Yes
Cridanimod	38609-97-1	Tox21	20.51133	82.39178	NA	NA	Yes
Dimethisoquin hydrochloride	2773-92-4	Tox21	11.53437	19.73928	NA	NA	Yes
Falnidamol dihydrochloride	1216920-18-1	Tox21	13.44809	44.78139	NA	NA	Yes
Lacidipine	103890-78-4	Tox21	23.01409	17.59346	NA	NA	Yes
Lintitript	136381-85-6	Tox21	0.258222	17.89915	NA	NA	Yes
Methyl 3-amino-5,6-dichloropyrazine-2-carboxylate	1458-18-0	Tox21	15.089	51.31506	NA	NA	Yes
Mitoxantrone	70476-82-3	Tox21	0.425266	17.54468	B-cell lymphoma cell line	GSE60408	Yes
Thioridazine	50-52-2	Tox21	23.91446	14.72563	PC-3 Cell Line, 9.8uM, 10uM	GSE5258	Yes
Thioridazine hydrochloride	130-61-0	Tox21	25.39531	16.8285	NA	NA	Yes
Triamterene	396-01-0	Tox21	23.91446	20.18841	NA	NA	Yes
Vincamine	1617-90-9	Tox21	34.66543	13.88488	PC-3 Cell Line, 11.2uM	GSE5258	Yes

All exposures were for 6 hrs at 50uM.

^1^Data from RT-qPCR studies in Fig 7.

Chemicals had to induce either *CXCL1* or *IL6* in wild-type cells but not NFKB1-null cells to be classified as confirmed.

HeLa cells were exposed to either 15 chemicals identified in the Tox21 screen or the 17 chemicals identified in the ToxCast screen and expression of *CXCL1* or *IL6* genes were examined (**S11 Table in** S1 File). All of the Tox21 chemicals except aminoquinuride activated *CXCL1* in wild-type cells that was abolished in similarly treated *NFKB1*-null cells (Fig 7B). Some of the same chemicals also activated *IL6* in wild-type cells. A subset of these chemicals exhibited abolishment of induction in the *NFKB1*-null cells. Similarly, all 18 of the ToxCast chemicals induced the *CXCL1* gene expression in wild-type cells that was uniformly abolished in the *NFKB1*-null cells (Fig 7C). A similar pattern was also observed for the *IL6* gene for most of the chemicals. Thus, the RT-qPCR studies indicated that almost all of the chemicals selected for validation induced NF-κB target genes in a *NFKB1*-dependent manner.

### Transcript profiling of chemicals in wild-type and NFKB1-null cells

**Fig 8:**
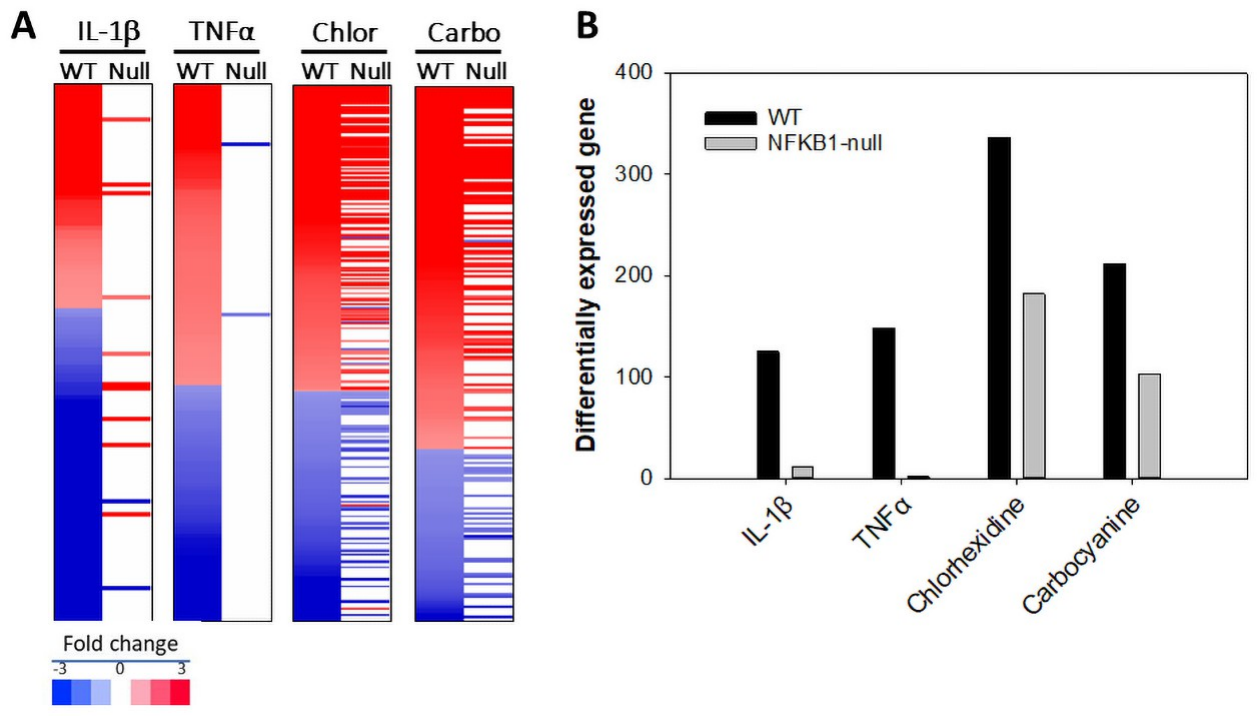
Transcript profiling of chemicals in wild-type and *NFKB1*-null cells. The indicated treatments were analyzed by TempO-Seq human S1500+ platform examining the expression changes in ~3000 genes. Significant expression changes were identified as described in the Methods. (A) The heat maps show the genes altered by the indicated treatment in wild-type HeLa cells and their expression after treatment in the *NFKB1*-null cells. Chlor, chlorhexidine diacetate; Carbo, carbocyanine. (B) Number of genes significantly altered in each treatment described in A.

In future HTTr studies, we propose that the use of cell lines nullizygous for important targets of environmental chemicals will facilitate the interpretation of gene expression patterns. To provide proof of this concept, we generated transcript profiles of activators of NF-κB in wild-type and *NFKB1*-null cells generated by TempO-Seq targeted sequencing of ~3000 human genes [55]. Fig 8A shows the heat maps of the filtered changes altered by IL1β, TNFα, carbocyanine and chlorhexidine diacetate in the two cell lines. Fig 8B shows the number of genes altered by each treatment in wild-type cells and the number of those genes that are altered in the *NFKB1*-null cells. Almost all of the genes regulated by IL1β and TNFα in wild-type cells were no longer regulated in the *NFKB1*-null cells. For carbocyanine and chlorhexidine, about half of the genes (49% and 54%, respectively) regulated in the wild-type cells were no longer regulated in the *NFKB1*-null cells. These studies demonstrate that many of the changes in gene expression after exposure to two putative NF-κB activators were *NFKB1*-dependent.

## Discussion

Gene expression biomarkers can identify chemical modulators of transcription factors, as demonstrated by previous work from our group and others [23, 56–59]. In the present study, we developed a biomarker to predict the modulation of NF-κB in response to environmental perturbations. We used published gene expression datasets with treatments known to activate NF-κB to demonstrate that our approach is a reliable method to predict activation of NF-κB.

We first identified genes that are NF-κB-regulated by analyzing published archived gene expression data from TNFα-stimulated HeLa cells with active or inactive NF-κB signaling controlled by overexpression of IκB. A weight of evidence strategy identified 108 genes consistently activated or repressed by TNFα at three time points in cells with active NF-κB signaling but not in its absence. The resulting biomarker consisted of 63 up-regulated and 45 down-regulated genes many of which are known direct targets of NF-κB, including *IL6* [30], *ICAM1* [31], and *IRF1* [32]. Genes in the biomarker with well-characterized functions within the NF-κB regulatory network included members of the TRAF protein family, interleukin (IL) cytokines, and members of the Rel/NF-κB family (NF-κB subunits encoded by *NFKB2* and *RELB*). The biomarker also included high ranking genes that are not apparently known NF-κB targets but play important roles in inflammatory responses. For example, the gene with the highest average fold-change in the biomarker was *EFNA1* (ephrin A1), which responds to TNFα through JNK and p38 MAPK signaling pathways that can be independent of NF-κB [60]. However, it should be noted that while TNFα regulates genes through both NF-κB-dependent and -independent mechanisms, the genes that were identified would be NF-κB-dependent because of the genetic filter imposed, i.e., IkB-dependent. The genes in the biomarker are most likely regulated by NF-κB through the canonical pathway. The ability of the biomarker to identify activation of the nonclassical pathway could not be thoroughly tested due to the lack of appropriate biosets.

Using biosets with treatments known to activate NF-κB, the biomarker had a predictive balanced accuracy of 90.3%. Given this high reliability, we then used the biomarker to identify chemical modulators of NF-κB in a large human microarray compendium [22]. This analysis identified 215 chemicals that were positively correlated and 49 chemicals that were negatively correlated with the NF-κB biomarker. We used the top 20 ranking biosets that resulted in NF-κB activation to examine published experimental links to NF-κB activation. For example, we found evidence that NF-κB is activated by crocidolite asbestos [3, 41] (Janssen, Driscoll et al. 1997) [3], nickel chloride [42], and curdlan [4, 43] (Rand, Robbins et al. 2013) [4]. For the 49 chemicals identified as putative inhibitors, many fell into five major groups according to their known molecular targets: 1) inhibitors of RelA and IKKα/β, 2) aurora kinase inhibitors, 3) inhibitors that act upstream of NF-κB on RAF, MEK, and P38a/b, 4) bromodomain (BRD) and extra terminal protein (BET) inhibitors, and 5) glucocorticoid receptor agonists.

Two HTS for NF-κB activators were carried out using cell-based, reporter gene assays. We selected a total of 32 chemicals predicted to activate NF-κB from the HTS assays, and we tested the ability of these 32 chemicals to activate NF-κB-dependent genes in wild-type and *NFKB1*-null HeLa cells. These studies demonstrated that wild-type vs. null cell line comparisons can unequivocally identify targets of environmental chemicals. To our knowledge, this is one of the first studies (in addition to Jackson et al. [6, 25] (Jackson AC 2020) [6] to compare profiles of chemicals between wild-type and nullizygous cell lines. Although the study was limited in scope, it provides support for the use of nullizygous cell lines in HTTr screening, especially in targeted screening to confirm predicted targets from primary HTTr screens. These findings also underscore the utility of multi-pronged screening approaches that include HTS assays, *in silico* screens, and targeted functional follow-up. The limited overlap between chemicals identified in the biomarker screen and those identified in HTS assays suggests that identification of NF-κB modulators is constrained by experimental approach. For example, thioridazine was identified as an NF-κB activator in the Tox21 chemical library and was identified in the screen of our compendium using the biomarker, but this chemical was “inconclusive” in the Tox21 follow-up assay. This discrepancy highlights the need for multiple lines of evidence, as the detection of transcription factor activity depends on a variety of variables, including cellular conditions (e.g., cell line and environment) and experimental approach.

In summary, the results of our screen demonstrate that our biomarker strategy can be used to readily identify NF-κB modulators. Despite extensively documented importance of NF-κB in gene-regulatory networks, this study provides the first gene expression biomarker for predicting the modulation of NF-κB in multiple cell lines. These results indicate that the NF-κB biomarker will be useful in analyzing HTTr data, such as screening of environmental chemicals in ToxCast libraries.

## Methods

### Use of a gene expression microarray experiment compendium

As described previously [59], annotation data from BaseSpace Correlation Engine (BSCE) (https://www.illumina.com/products/by-type/informatics-products/basespace-correlation-engine.html; formerly NextBio) was used to build a spreadsheet of gene expression comparisons (called biosets) from experiments carried out using human cell lines and tissues. This compendium included the study accession information, bioset name, cell line, tissue, chemical name and in many cases, chemical concentration and treatment time. Of the biosets included, 12,061 biosets were derived from experiments from chemically treated cells. Methods used to derive the filtered gene lists are described in detail in Kuperschmidt et al. [22]. In short, gene expression data were processed using BSCE protocols to generate filtered gene lists with expression fold-change values for each bioset; information about the methods used to normalize and identify differentially expressed genes are found in Kuperschmidt et al. [22]. Gene lists were filtered to include only genes which had an absolute fold change magnitude of ≥1.2 and p < 0.05.

### Construction of the NF-κB biomarker

The NF-κB biomarker was derived from raw microarray expression data from Tian et al. [29] (GSE2624 available on the NCBI GEO database). Genes were selected that were differentially expressed in the same direction in at least 2 of the 3 biosets from the TNFα-treated wild-type cells but not in the same direction from TNFα-treated cells overexpressing IκB. Genes were further filtered for an average fold change across those biosets in the wild-type cells which showed significant expression of at least 1.5-fold in either direction. The final biomarker consisted of 108 genes and average fold-change levels. This biomarker was uploaded to BSCE for subsequent analyses.

### Ingenuity pathway analysis

The NF-κB biomarker genes were analyzed using the canonical pathway and upstream analysis functions of Ingenuity Pathway Analysis (IPA, Qiagen Bioinformatics, Redwood City, California). IPA was used to calculate the significance of overlap of the biomarker genes with canonical pathways and upstream transcription factors using a right-tailed Fisher’s Exact test, yielding a list of p-values describing the probability of the overlap between the NF-κB biomarker gene list and the IPA pathway gene lists. Upstream analysis used the number of differentially expressed genes to predict upstream regulators of the biomarker genes.

### Comparison of the biomarker to database biosets

The NF-κB biomarker was compared with all other biosets in the database using the Running Fisher algorithm. This method provides an assessment of the statistical significance of the overlapping genes between the biomarker and each bioset by assessing correlation and providing a summary p-value. A complete description of the Running Fisher test is provided in Kuperschmidt *et al*. [22]. While we acknowledge that other methods have been used to make comparisons between two gene lists (such as in GSEA), the Running Fisher test is the only method provided in BSCE to compare the gene lists. The Running Fisher test has worked remarkably well for the prediction of transcription factor modulation in mice, rats and humans [7–9]. The p-values of the pair-wise comparisons were exported and converted to a -log(*p*-value), with negative values used to indicate negative correlation between the biomarker and the bioset. Biosets with |-log(*p*-value)| ≥ 4 were considered significant based on prior studies using this threshold [23, 56, 58]. A column in the human gene expression spreadsheet was populated with the -log(*p*-value) for each bioset. Biosets that were positively correlated with the biomarker (-log(*p*-value) ≥ 4) were predicted to exhibit activation of NF-κB; biosets that were negatively correlated (-log(*p*-value) ≤ -4) were predicted to exhibit suppression of NF-κB.

### Selection of positive and negative controls and calculation of biomarker accuracy

In the database of human gene expression comparisons, biosets were identified that examined the effects of immunomodulator factors on global gene expression in human cell lines. Biosets examining the effect of more than one factor or that could not be interpreted were not used. For example, the bioset “Colorectal cancer HT 29 cells 24hr IFNG and GATA6L overexpression—2hr TNF _vs_ no TNF” derived from the study GSE72079 compared the effects of a 2hr treatment with TNFα. Since both the treated and control cells were treated with interferon gamma and overexpressed the long form of the gene *GATA6*, this study was eliminated from consideration. The resulting list of biosets were not filtered for time of exposure or concentration of the factor used in the experiment, although it is acknowledged that these are important parameters to consider in evaluating the value of the comparison as a true positive or true negative. The 6 biosets used to create the biomarker which examined TNFα effects (essentially the training set) were not included in this list. The accuracy analysis was carried out two ways. In the first method, true positive biosets were used individually, assuming that the conditions of exposure would always lead to activation of NF-κB. However, there were a number of time course studies in which NF-κB was activated or suppressed during only part of the time window of exposure (see Results). Thus, the second method examined the true positive biosets by study. If any bioset in the time course study was positive for NF-κB activation, then the study was called positive. By individual bioset, there were 43, 90, and 79 true positives according to the–(log(p-values)). Using the method based on study ID, there were 25, 56, and 47 true positives for IL1α/β, LPS, or TNFα, respectively. True negatives were selected from the database based on the fact that a number of immunomodulatory factors (IL2, IL3, IL4, IL6, IL12, IFNα, and IFNβ) were not expected to activate NF-κB. There were a total of 208 biosets which were classified as true negatives (**S4 Table in** S1 File). These biosets were also evaluated both individually and by study. The values for predictive accuracy were calculated as follows: sensitivity (true positive rate) = TP/(TP+FN); specificity (true negative rate) = TN/(FP+TN); positive predictive value (PPV) = TP/(TP+FP); negative predictive value (NPV) = TN/(TN+FN); balanced accuracy = (sensitivity+specificity)/2.

### High-throughput screening for NF-kB activators in the Tox21 chemical library

Tox21 data were accessed through the Tox21 Data Browser (https://tripod.nih.gov/tox21/). Data were downloaded for the NF-κB assay (“tox21-NF-κB-bla-agonist-p1”), which is a cell-based, ratiometric readout assay performed using ME-180 human cervical cancer cells. Compounds with the “active agonist” call as the assay outcome were considered positive hits [10]. It is acknowledged that the compounds are likely not directly interacting with NF-κB but are activating indirectly. Because this assay relied on fluorescence signals from a β-lactamase reporter system, autofluorescent compounds may result in false positives. Autofluorescent compounds were identified and filtered using the “flag” field. Fifty-five unique compounds were identified as activators, and 31 of these passed the filter for autofluorescence (**S7 Table in** S1 File). We compared the list of 55 hits to the compounds revealed in the screen of our microarray compendium and identified 3 compounds in four biosets that overlapped as hits in both the Tox21 data and our compendium. Two of these compounds were in the set of 31 agonists that appeared promising for further validation. One compound (benzo(a)fluoranthrene) of the 55 was flagged as autofluorescent but was included in the validation set. Based on availability, 26 of the 32 identified agonists were selected for further validation using the same assay. Out of the 26 chemicals examined, 15 chemicals were confirmed as NF-κB activators in a secondary screen. Fourteen of the 15 chemicals were evaluated by RT-qPCR. An additional chemical, thioridazine, which was “inconclusive” in the Tox21 follow-up assay, was also examined as that chemical was identified in the screen of our compendium using the biomarker.

### High-throughput screening for NF-κB activators in the ToxCast chemical library

Activation of NF-κB was determined in a multiplexed reporter gene assay encompassing 48 transcription factor binding sites including one for NF-kB in the human cell line HepG2 as previously described [11]. The endpoint was queried for active chemicals from a total of 3806 samples using the assay endpoint name ATG_NF_kB_CIS_up (all results available at https://comptox.epa.gov/dashboard/assay_endpoints/ATG_NF_kB_CIS_up). Initially, 165 samples with active hit calls were identified and subsequently prioritized to 17 based on robustness and potency of concentration-response curves for further characterization (**S8, S9 Tables in** S1 File).

### Culture and treatment of wild-type and NF-κB-null cells

Chemicals were obtained through the Tox21 or ToxCast chemical procurement programs and were ≥ 95% pure. All chemical stock solutions were supplied in DMSO. TNFα recombinant human protein (≥95% purity) was obtained from Thermo Fisher/Gibco (Carlsbad CA), IL1β came from Thermo Fisher/Life Technologies (Frederick, MD). A *NFKB1* nullizygous cell line engineered in HeLa cells using in part CRISPR/Cas9 technology was obtained from Edigene, along with the wild-type HeLa cell line. Cells were cultured in DMEM media (GIBCO) supplemented with 10% FBS (Omega Scientific, Australia) and 1x penicillin/streptomycin/glutamine. Cells were plated at 8 x10^5 cells per well in 24-well plates. After 20 hours, media was replaced with dosing solutions containing DMSO (0.05%), IL1β (1 ng/mL), TNFα (5 ng/mL), or the Tox21 and ToxCast chemicals (50 μM) in wild-type and the *NFKB1*-null cells. After 6 hours of exposure, media was removed, and cells were lysed in 0.3 mL Trizol, followed by RNA extraction.

### Evaluation of gene expression by RT-qPCR

Gene expression was quantified using reverse transcription quantitative PCR (RT-qPCR). Total RNA was reverse transcribed with the SensiFAST cDNA Synthesis Kit per manufacturer instructions (Bioline). cDNA was then amplified in 384-well PrimePCR assay plates (Bio-Rad) with Sso Advanced Universal SYBR Green Supermix (Bio-Rad). Primers were designed using Primer3 v0.4 [61] and are listed in **S10 Table in** S1 File.

### Evaluation of gene expression using TempO-Seq

Gene expression in wild-type and *NFKB1*-null cells was evaluated for gene expression changes after exposure to IL1β, TNFα, carbocyanine and chlorhexidine diacetate at the same concentrations as described above using the human S1500+ Tempo-Seq platform [55] (BioSpyder, Inc, Carlsbad, CA). After extraction, RNA samples were sent to BioSpyder for analysis. Raw read counts were normalized and filtered gene lists (p-value < 0.05 with no multiple test correction) were generated using the DESeq2 module in Partek Flow. The data is publicly available at Gene Expression Omnibus, accession number GSE153616. (Reviewers: to be released once manuscript is accepted).

## Supporting information

S1 File (XLSX)

S2 File (DOCX)
