# Supplementary material for: A gene expression biomarker for predictive toxicology to identify chemical modulators of NF-κB
Source: PLoS One. 2022 Feb 2;17(2):e0261854. doi: 10.1371/journal.pone.0261854 (PMC8809623; doi:10.1371/journal.pone.0261854)
Supplement: S2 File — (DOCX) [file pone.0261854.s002.docx]

**Supplemental File 2**

**For**

**Identification of Chemical Modulators of NF-κB Using a Gene Expression Biomarker Approach**

Katharine L. Korunes^1,2,3^, Jerry Liu^1^, Ruili Huang^4^, Menghang Xia^4^, Keith Houck^1^, and J. Christopher Corton^1,*^

^1^Center for Computational Toxicology and Exposure, US Environmental Protection Agency, Research Triangle Park, NC 27711

^2^Biology Department, Duke University, Durham, NC 27708

^3^Present address: Department of Genetic Anthropology, Duke University, Durham, NC 27708

^4^National Center for Advancing Translational Sciences, National Institutes of Health, Bethesda, Maryland 20892

**
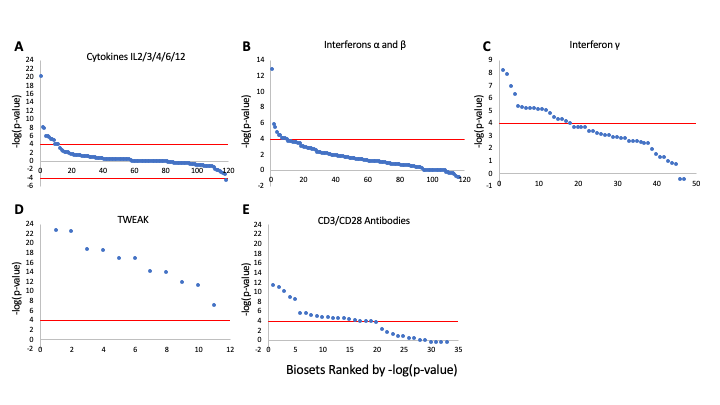
**

**Figure S1. Effects of immunomodulatory factors on NF-κB activity.**

Biosets derived from cells exposed to a number of immunomodulator factors were assessed for effects on NF-κB.

A. Results following treatment with cytokines IL2, IL3, IL4, IL6, or IL12.

B. Effects of treatment with interferons α and β.

C. Effects of treatment with interferon γ.

D. Biomarker behavior following treatment with cytokine tumor necrosis factor-like weak inducer of apoptosis (TWEAK).

E. Results following cotreatment with antibodies against CD3 and CD28.


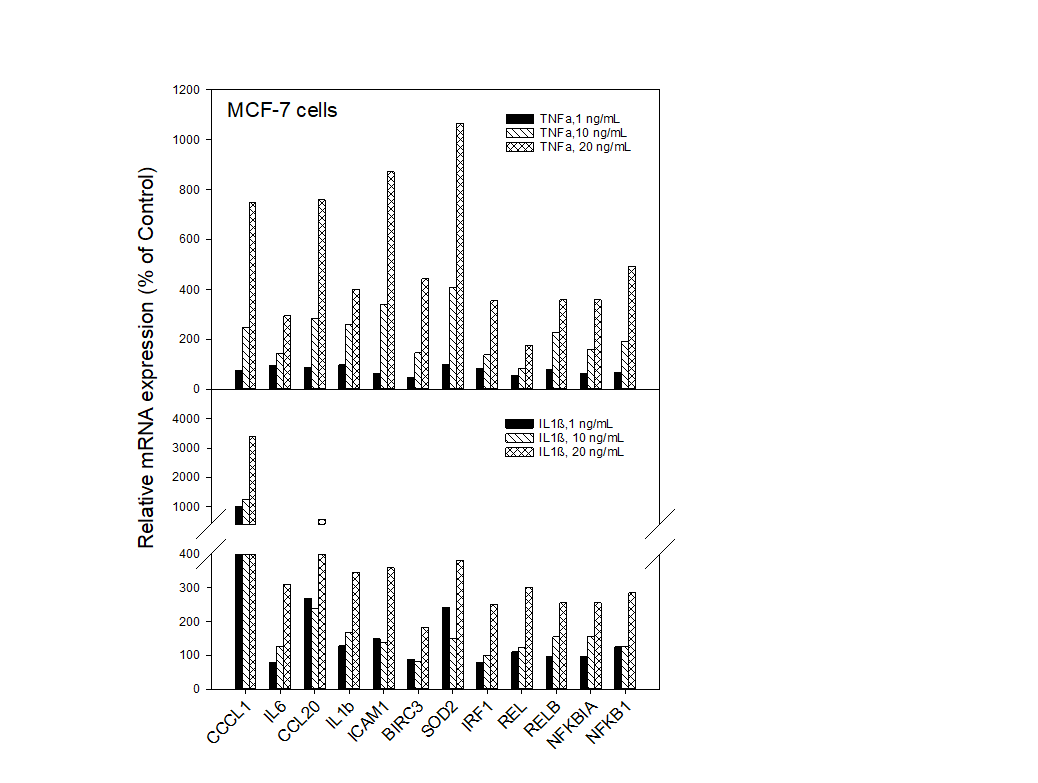


**Figure S2. Expression of NF-κB biomarker genes after exposure of MCF-7 cells to TNFα and IL1β.**

MCF-7 cells were exposed to TNFα or IL1β at the indicated concentration for 6 hours. RNA was isolated and RT-qPCR was used to determine the expression levels of the indicated genes.
